# Contributions of epithelial-mesenchymal transition and cancer stem cells to the development of castration resistance of prostate cancer

**DOI:** 10.1186/1476-4598-13-55

**Published:** 2014-03-12

**Authors:** Ping Li, Ru Yang, Wei-Qiang Gao

**Affiliations:** 1 State Key Laboratory of Oncogenes and Related Genes, Stem Cell Research Center, Renji Hospital, School of Medicine, Shanghai Jiao Tong University, Shanghai, China; 2 Med-X Research Institute, Shanghai Jiao Tong University, Shanghai, China

**Keywords:** Castration-resistant, Prostate cancer, Epithelial-to-mesenchymal transition, Cancer stem cells, Signaling pathways

## Abstract

An important clinical challenge in prostate cancer therapy is the inevitable transition from androgen-sensitive to castration-resistant and metastatic prostate cancer. Albeit the androgen receptor (AR) signaling axis has been targeted, the biological mechanism underlying the lethal event of androgen independence remains unclear. New emerging evidences indicate that epithelial-to-mesenchymal transition (EMT) and cancer stem cells (CSCs) play crucial roles during the development of castration-resistance and metastasis of prostate cancer. Notably, EMT may be a dynamic process. Castration can induce EMT that may enhance the stemness of CSCs, which in turn results in castration-resistance and metastasis. Reverse of EMT may attenuate the stemness of CSCs and inhibit castration-resistance and metastasis. These prospective approaches suggest that therapies target EMT and CSCs may cast a new light on the treatment of castration-resistant prostate cancer (CRPC) in the future. Here we review recent progress of EMT and CSCs in CRPC.

## Introduction

Prostate cancer is the leading cause of cancer incidence and the second leading cause of cancer-related deaths amongst males in the United States. Androgen deprivation therapy (ADT) remains the mainstay therapy for advanced prostate cancer in addition to surgery. Many studies have been focused on androgen/androgen receptor (AR) signaling axis. Drugs targeting AR pathway have been developed in the past several decades, including estrogens (such as diethylstilbestrol, a luteinizing hormone-releasing hormone inhibitor), steroidal anti-androgens (such as cyproterone acetate, megestrol acetate and medroxyprogesterone acetate) and nonsteroidal antiandrogens (such as flutamide, nilutamide and bicalutamide, androgen receptor blockers), and gonadotropin-releasing hormone (GnRH) antagonists (such as abarelix and degarelix).

Although initially effective at regressing tumor growth, these androgen deprivation therapies will ultimately fail, rendering a lethal drug-resistant stage commonly known as castration-resistant prostate cancer (CRPC). The biological basis underlying the development of metastatic androgen-independent prostate cancer has been addressed, mainly focusing on AR over-expression, mutation, and cross-talk to other growth factor signaling pathways [1]. Due to recognition of continued AR signaling in the progress of CRPC, several new drugs include MDV3100, abiraterone acetate and VN-124-1 have been investigated in clinical trials, and the former two drugs have been approved by US Food and Drug Administration last year. However, the potential mechanisms by which prostate cancer cells become CRPC still remain largely unclear.

**Figure 1:**
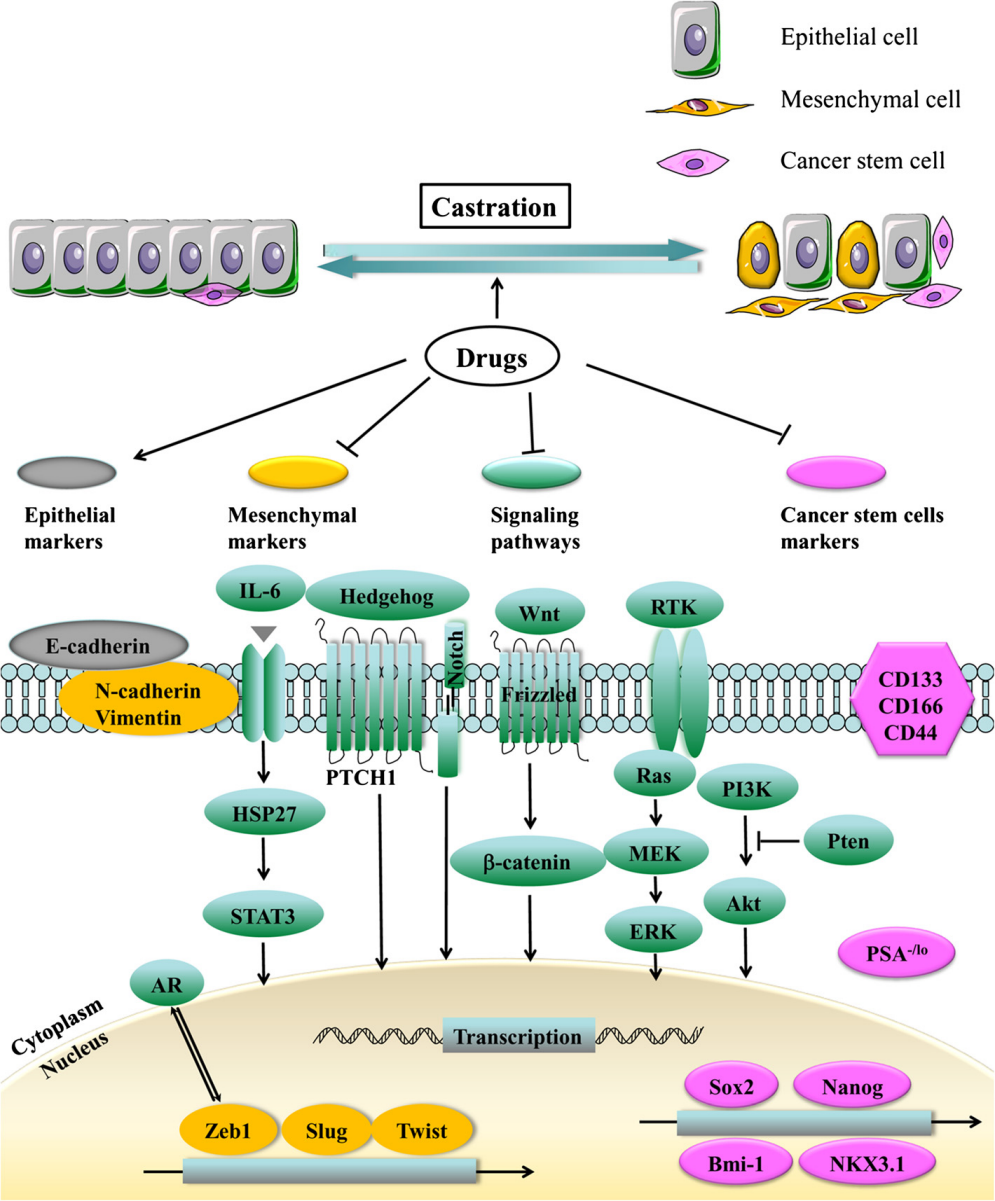
**Contribution of EMT, CSC and related signaling to CRPC.** Castration induces EMT and enhances the stemness of CSCs, which are the two key contributors for the development of castration resistance. Following castration, epithelial cells lose their epithelial phenotypes, i.e., biomarkers such as E-cadherin, but gain mesenchymal characteristics or biomarkers such as N-cadherin and Zeb-1. Cancer stem cells are stem cell-like tumor cells, which have the capability of self-renewal and differentiation. Castration can enrich this population. Certain molecules might be regarded as prostate cancer stem cell markers, such as CD133, CD166, and etc. Specific signaling including Wnt, Notch, SHH, and others might be the underlying molecular basis of the functions of EMT and CSCs. The EMT appears to be a dynamic process. Development of drugs either inhibiting EMT, CSCs and specific signaling pathways or enhancing expression of epithelial cell markers might be a novel, additional strategy to treat CRPC in the future. (→, Promote; ⊥, Inhibit.)

In recent years, accumulating evidences indicate that epithelial-to-mesenchymal transition (EMT) and cancer stem cells (CSCs) play important roles during the development of drug resistance of prostate cancer. Unveiling the molecular mechanisms responsible for EMT and CSCs would help to develop new promising therapies for metastatic prostate cancer in the future. In this review, we will summarize our current understanding regarding EMT and CSCs in CRPC, including possible relationships between EMT and CSCs and specific signaling pathways involved during generation of EMT and CSCs (Figure 1).

### Epithelial-to-mesenchymal transition and castration-resistant prostate cancer

EMT has been known to be involved in a spectrum of physiology and pathology process. Originally EMT is a physiological process in which epithelial cells turn into mesenchymal cells through a specific signaling pathway. Via EMT, epithelial cells lose their epithelial phenotypes such as cell polarity and cell-cell adhesion, and gain mesenchymal characteristics such as high capability of migration, invasion, anti-apoptosis and disorganization of extracellular matrix. Prostate tumors are adenocarcinomas, arising originally from the glandular epithelium of the prostate gland and prostatic ductules. Castration can induce EMT [2], which might be a critical biological process for the malignant tumor cells of epithelial origin to leave the epithelium, invade into the stroma area, and disseminate to distal organs, as reported in recent studies [3-5].

Multiple proteins participate in EMT and its reverse process, MET (Mesenchymal-epithelial transition). A variety of biomarkers have been demonstrated for studies on EMT, including E-cadherin, N-cadherin, Vimentin, Snail, Zeb1, Twist and others [6]. Amongst them, E-cadherin locates in the cell surface of epithelial tissues and mediates cell-cell adhesion to bind cells together in the normal epithelial cells. Its expression level is negatively correlated with the occurrence of EMT and tumor invasion. On the contrary, Vimentin (a cytoskeletal marker) and N-cadherin (a cell surface marker) are associated with the initiation of EMT and the progression from well differentiated adenoma to invasive carcinoma. In addition, Snail, Slug, Zeb1, Zeb2 and Twist are able to down-regulate the level of E-cadherin and drive EMT to occur [7].

Sun et al [2] have found that androgen deprivation results in EMT in the prostate cancer. Epithelial marker (e.g. E-cadherin) levels are decreased whereas mesenchymal marker (e.g. N-cadherin, Zeb1, Twist1, and Slug) expression is increased in normal mouse prostate tissue following castration. Similar changes are observed in castrated human LuCaP35 xenograft tumors. They also prove that an EMT occurs in human samples undergoing ADT. During the EMT transition, a mesenchymal marker Zeb1, a transcription factor, mediates the progress by Zeb1‒AR feedback loop [2]. The evidence for Zeb1 to be involved in CRPC has also been supported by another group. Graham et al find that Zeb1 is markedly enhanced in prostate cancer cells and insulin-like growth factor-I (IGF-I) is responsible for the overexpression of Zeb1 [8]. Twist, another transcription factor, is highly expressed in prostate cancer and twist expression is strongly associated with Gleason score. Blockade of Twist increases the level of E-cadherin and decreases the capability of invasion and migration of androgen-independent prostate cancer cells [9,10]. In addition, Slug, another EMT transcription factor, is regulated by androgen, cooperates with AR and promotes the development of CRPC [11].

Similarly, Reiter and colleagues have found a remarkable increase in N-cadherin expression in CRPC xenografts as well as in both primary and metastatic tumors of patients with CRPC [12]. Application of exogenous N-cadherin could induce EMT, invasion, migration of multiple prostate cancer cell lines *in vitro* and *in vivo*. Specific N-cadherin antibody could suppress the process of EMT, decrease tumor growth, invasion and migration, and block the progression to castration-resistance via reducing the activity of AKT and IL-8 expression. Therefore, this group has identified N-cadherin as a critical cause of prostate cancer metastasis and CRPC. It is proposed that therapies targeting this EMT component with monoclonal antibodies will be a promising approach for further preclinical and clinical validation.

Meanwhile, several EMT biomarkers have been demonstrated to be associated with the development of prostate cancer in the last decade. Loss of E-cadherin or switch of E-cadherin to N-cadherin leads to destruction of cell-cell adhesion, which drives adenoma to become carcinoma [13]. As an osteoblast cadherin, Cadherin-11 could provide association between prostate cancer cells and osteoblasts, enhance the invasion and migration capacities [14]. Zinc-finger transcription factor Snail could not only repress the transcription and expression of E-cadherin but also trigger EMT in prostate cancer [15]. Although these molecules have been investigated for prostate cancer in general and not specific for CRPC, they might be potential candidates of EMT markers for CRPC.

In short words, these studies together suggest that EMT and its biomarkers contribute to drug resistance in prostate cancer [16]. E-cadherin, N-cadherin, Zeb1, Twist, Slug, Snail and other EMT markers play important roles in the regulation of the invasive and metastatic potential of prostate cancer cells. Therefore, therapeutic strategies purporting to intervene EMT process or to reverse EMT phenotypes might become alternatives for future cancer therapy.

### Cancer stem cells and castration-resistant prostate cancer

CSCs are proposed to be stem-like cells in tumors, which have the ability to self-renew and to differentiate into new diverse tumor cells. These cells are thought to be a subpopulation of the tumor cells that express specific surface antigens and possess mesenchymal phenotypes, which are important in tumor initiation and progression including castration resistance and metastasis.

As an important mechanism in the *de novo* theory of CRPC, CSCs are referred to as malignant epithelial stem cells in the lurker cell pathway [1]. Very early, John Isaacs [17] has postulated that initial occurrence of a subpopulation of androgen-independent tumor cells can cause the fail of androgen ablation therapy and the development of CRPC. Denmeade and colleagues [18] reveal that the basal cells of prostate contain a subpopulation of androgen-independent epithelial stem cells. In support of this hypothesis, using a novel human prostate cancer xenograft (LAPC-9), Craft et al [19] have reported that the occurrence of CRPC attributed to clonal expansion of a small percentage of androgen-independent cells. They conclude that prostate cancers contain both androgen sensitive and insensitive cells and selective pressure of ADT alters the relative frequency of these cells, leading to development of CRPC.

CSCs biomarkers are searched and used to identify and isolate CSCs in prostate cancer. Frequently used biomarkers in CRPC related CSCs include Nkx3.1, CD166, PSA^-/LO^, Nanog, Bmi-1 and Sox2 (Table 1). Other potential biomarkers contain Lgr4, Sca-1, α2β1, CD44, CD44^+^/α2β1^hi^/CD133^+^, CD44^+^ CD24^-^, p63, Lin^-^CD44^+^CD133^+^Sca-1^+^CD117^+^, Trop2, ALDH1 and others (Table 1). The features and related studies for each of the above mentioned markers are listed in Table 1.

**Table 1:** EMT markers, cancer stem cell markers and signaling pathways involved in EMT and CSC in prostate cancer, especially in castration-resistant prostate cancer

**EMT marker**	**Function**	**CRPC**	**Refs**
E-cadherin	Regulates the invasive capacity of prostate cancer cells		[3,57,58]
β-Catenin	Regulates the process of EMT and metastatic phenotypes		[59]
N-cadherin	Promotes growth, metastasis and castration resistance in prostate cancer	Yes	[12,60,61]
Cadherin-11	Enhances migration and invasion capacity of prostate cancer cells, increases the association with osteoblasts		[14,62]
Vimentin	Promotes prostate cancer cell invasion and metastasis	Yes	[63]
Fibronectin	Protects cells from undergoing apoptosis		[64,65]
Collagen 1	Have an effect on EMT of prostate cancer cells		[65]
alphaII(b)beta3 integrin	Participates in the metastatic progression of prostatic adenocarcinoma		[66]
Syndecan-1	Associates with Gleason score and tumor progression of prostate cancer		[67-69]
Zeb1	Altering the invasive phenotype of Prostate cancer cells	Yes	[2,8,70]
Slug	Correlates with advanced pathological grades of prostate cancer	Yes	[11,71]
Snail	Contributes to prostate cancer progression and metastasis		[4,5,72,73]
Twist	Correlates with Gleason grading and metastasis	Yes	[9,10]
ETS-1	Mediates by TGF-β, affects cell growth and tumor formation	Yes	[74,75]
**CSC markers**	**Function**	**CRPC**	**Refs**
Lgr4	Regulates early prostate development and stem cell differentiation		[32]
α2β1 integrin	Produces prostate-like glands		[30]
CD133	Functions as a normal prostate stem cell marker and has tumor formation ability		[76]
CD166	A potential surface marker for castration resistant tumor cells	Yes	[21]
PSA	Displays increased colony and sphere-form capacity	Yes	[22]
CD44	Associates with cells of neuro-endocrine phenotype		[77]
CD44^+^/α2β1^hi^/CD133^+^	Presents high proliferative ability *in vitro* and can differentiate to an AR–positive phenotype similar to prostate cancers *in vivo*		[78]
CD44+ CD24(-)	Exhibits stem cell characteristics and predicts overall survival in prostate cancer patients.		[79]
Sca-1	Have high proliferative ability and high capacity to reconstitute prostatic tissue		[31]
Nkx3.1	Indicates that luminal cells might be a cell of origin	Yes	[20]
p63	Produces all epithelial lineages of the adult prostate (i.e., basal, luminal, and neuroendocrine cells)		[80,81]
Lin^-^Sca-1^-^CD49f^+^ (LSC)	Produces prostatic tubule structures		[82]
Lin^-^CD44^+^CD133^+^Sca-1^+^CD117^+^	Produces a prostate after transplantation *in vivo*		[29]
Trop2	Trop2^hi^ basal cells give rise to basal, luminal, and neuroendocrine cells *in vivo*		[83]
ALDH1	Associates with a poor prognosis for patients with prostate cancer		[84,85]
Nanog	Promotes CSC phenotypes and properties *in vitro* and *in vivo*, promotes AI phenotypes and CRPC regeneration	Yes	[24,25]
Bmi-1	A key regulator of self-renewal activity, plays central roles in malignant progression of prostate cancer	Yes	[26]
Sox2	Inhibits by AR signaling and play an important role in CRPC	Yes	[28]
TRA-1-60, CD151 and CD166	Exhibits enhanced sphere-forming capacities *in vitro* and tumor-initiation capacities *in vivo*		[86]
**Signal pathway involved CSC and EMT**	**Function**	**CRPC**	**Refs**
AR	A key regulator for the acquisition of EMT phenotypes	Yes	[42,45]
PTEN/AKT	Promotes prostate tumor growth and metastasis		[87]
AKT/GSK-3β	Participates in TNFα-induced EMT process		[88]
ERK	Has a profound feedback on EGFR signaling		[89]
AKT	Has a great effect on cell migration via induction of the EMT characteristics		[89]
TGF-β	Associates with malignant progression of prostate cancer by activation of the EMT phenotypes		[90,91]
CCL2/CCR2-STAT3	Promotes prostate cancer cell migration/invasion and EMT pathways	Yes	[92]
Hsp27-STAT3-Twist	Promotes prostate cancer metastasis, regulates the process of EMT	Yes	[39]
PTEN and RAS/MAPK	Accelerates prostate cancer malignant progression accompanied by acquisition of EMT phenotypes and stem-cell like properties	Yes	[37]
NF-kappaB	Correlates with EMT in human prostate cancer cells and may be functionally associated with the stem-like human prostate tumor initiation cells		[86,93,94]
JAK-STAT	Participates in significantly different gene expression in prostate cancer stem cells		[95]
PDGF-D	Mediates EMT process and regulates cancer cell invasion		[96]
IGF-1	Regulates EMT associated migration and invasion via elevated Zeb1 expression	Yes	[8]
FGFR-1	Leads to an EMT and distant metastasis		[38]
EGFR	Presents loss of cell-cell junctions with decreased epithelial markers and enhanced mesenchymal markers		[89]
WNT	Mediates EMT phenotypes and stemness maintenance of prostate cancer cells	Yes	[40,97]
Notch and Hedgehog	Regulates drug resistance and plays important roles in malignant transformation	Yes	[98,99]
Hypoxia-ERβ-HIF-1a/VEGF-A	Mediates EMT and have an implication in Gleason grading		[100]
DAB2IP	Regulates EMT and prostate cancer metastasis and serves as a target gene of EZH2 in prostatic epithelium		[101-103]
p63/miR205	Suppresses cell migration and metastasis		[104]
	Produces changes in Golgi polarization		

Several biomarkers have been identified to be associated with the CSCs in CRPC. For example, Shen and colleagues [20] have found that castration-resistant Nkx3.1-expressing positive cells (CARNs), a subpopulation of luminal epithelial cells, are CSCs in their study of lineage association between normal prostate progenitor cells and cancer cells. Basal cells decrease and luminal cells proliferate in the oncogenic formation. Compared with control Nkx3.1Cre^ERT2/+^; Pten^+/+^ mice with normal phenotype, Nkx3.1^CreERT2/+^; Pten^flox/flox^ mice develop high-grade prostatic intraepithelial neoplasia (PIN) and carcinoma following inducible deletion of Pten in the Nkx3.1 population [20]. In addition to the homeobox-containing transcription factor Nkx3.1, some cell surface markers are always available for identifying CSCs in both murine and human prostate tissues. One of the surface markers, CD166, is identified as a potential surface marker for castration resistant tumor cells [21]. The level of CD166 increases in both murine castrated prostate epithelial cells and human CRPC. CD166^hi^ population isolated by CD166 marker has higher capacity to form tumor-spheres, compared with CD166^lo^ population. In addition, compared to TROP2^hi^CD49f^hi^CD166^lo^, TROP2^hi^CD49f^hi^CD166^hi^ subset detects increased regeneration capacity *in vivo*. Over-expression of CD166 and CD166^hi^ cells is correlated with castration resistance in both Pten deleted mice and human prostate cancer cells [21]. Interestingly, PSA^−/lo^ prostate cancer cells are also demonstrated as an important cell type for CRPC [22]. Compared with PSA^+^ cells, PSA^−/lo^ prostate cancer cells are more clonogenic and tumorigenic. They resist to castration, form holoclones and spheres and develop tumors. PSA^−/lo^ prostate cancer cells are enriched following ADT and initiate a new outbreak of tumor development. A decrease in the number of PSA-producing cells is also observed by another group in patients after ADT [23].

Of note, a couple of pluripotent stem cell markers have also been associated with CRPC cells, further raising the possibility that stem cells play critical roles in the progression to a castration-resistant condition. For example, Nanog facilitates the tumor cells to acquire CSCs phenotypes and properties and promotes androgen independence in androgen deprived environment. LNCaP cells with over-expression of Nanog are easier to agglomerate together to form clones and spheres *in vitro* and lead to tumor *in vivo* following castration [24,25]. Similarly, Witte and colleagues have also reported that Bmi-1 mRNA level is enhanced in castrated mice prostate tissues and it maintains the stemness of p63^+^ stem cells. Suppression of Bmi-1 slows down the progression of malignant tumors in Pten-deletion prostate cancer model [26]. Moreover, a recent study shows that Sox2 is a critical regulator in self-renewal and tumor progression of human prostate cancer [27]. In addition, Sox2 could also be suppressed by AR and closely associated with castration-resistant tumor growth [28].

As many researchers believe that CSCs may arise from the gene mutations in normal stem cells, it is important to identify markers for stem cells in normal tissues. In this regard, a couple of stem cell markers are recently identified in normal prostates in addition to the above mentioned biomarkers in CRPC. Gao and colleagues have generated a functional prostate gland from Lin^-^CD44^+^CD133^+^Sca-1^+^CD117^+^ stem cells [29]. Based on α2β1, stem cells of human prostate epithelial are identified and isolated by Collins and colleagues [30]. A study from Burger et al indicates that prostate stem cell antigen Sca-1, a cell surface marker, is over-expressed in proximal regions of prostatic ducts. Sca-1 cells purified from the proximal ducts have higher capacity of proliferation [31]. Another recent study shows that Lgr4 regulates both prostate epithelial stem cell differentiation and prostate development [32]. Because the stemness of stem cells is linked to tumorigenesis, the cells expressing stem cell markers may be the origin for cancer. The finding of these stem cell markers in the development of prostate may provide a potential therapeutic target for prostate cancer.

Altogether, CSCs are considered to be a novel theory to elucidate the mechanism of surviving prostatic tumor cells following castration. The biomarkers of CSCs could be potential targets for treatment of castration-resistant tumor cells. In conjunction with ADT, novel therapeutics targeting CSCs, e.g. Nkx3.1^+^, CD166^hi^, TROP2^hi^CD49f^hi^CD166^hi^, TRA-1-60/CD151/CD166, PSA^−/lo^, Nanog, Bmi-1 cells, might be developed to eradicate remaining refractory tumor cells and to prevent recurrence of CRPC.

### Possible link between epithelial-to-mesenchymal transition and cancer stem cells in castration-resistant prostate cancer

Numerous studies have shown that EMT and CSCs are primary mechanisms for drug resistance in cancer including CRPC. Recently, a few studies have shown that characteristics of EMT are closely associated with the signatures of CSCs, which could lead to tumor recurrence and drug resistance phenotype.

Mani et al [33] recently find the experimental evidence to connect EMT to the emergence of CSCs in breast cancer. They have demonstrated that after TGF-β treatment (a potential inducer of EMT), differentiated mammary epithelial cells give rise to CD44^high^ CD24^low^ stem-like cells, as is seen in the case of induced by the expression of well-known E-cadherin transcription repressors, such as Twist and Snail. Very importantly, a recent elegant study by Weinberg group indicates that Zeb1, a key EMT regulator, is sufficient to switch the cells from a non-cancer stem cell to a cancer stem cell status and is required for the maintenance of the stemness of breast cancer stem cells [34]. Similar phenomenon is observed in pancreatic cancer. ZEB1, a signature of EMT, suppresses miR-200c, miR-203 and miR-128, which inhibit pluripotency genes, such as the Bmi-1, Sox2 and Klf4 genes [35]. These studies have made a link between EMT and CSCs stemness in breast cancer and pancreatic cancer and raise a possibility that EMT and CSCs can contribute either alone or in conjunction with each other to the initiation and progression of various types of cancers, including prostate cancer. As expected, Kong et al [36] further report that PC3 prostate cancer cells which are forced to express PDGF-D display EMT characteristics and show cancer stem-like cell features after over-expression of pluripotency genes, such as the Nanog, Oct4, Sox2, Lin28 and activation of polycomb repressor complex, which is associated with increased clonogenic and prostasphere-forming capacity *in vitro* and tumorigenicity *in vivo*. During the process, miR-200b and miR-200c play a critical role in linking EMT phenotypes and CSCs signatures. Over-expression of miR-200 family leads to reversed EMT as well as suppressed self-renewal ability by regulating Notch1 and/or Lin28B expression [36]. Sun et al [2] have also presented the evidence that the association between EMT induction and the emergence of prostate CSC-like phenotype following androgen deprivation. By comparison of the gene expression profiles of the prostate tissues from normal mice to castrated mice, a dramatic decrease in E-cadherin expression and an induction of the expression of mesenchymal markers such as N-cadherin, Zeb1, Twist and slug are observed after castration. Microarray gene analysis reveals that several mesenchymal markers, including Vimentin, Zeb1, Zeb2, Twist1, Snail1 and Slug are significantly increased in the Lin^-^CD44^+^CD133^+^Sca-1^+^CD117^+^ stem cells, as compared to those Lin^-^CD44^-^CD133^-^Sca-1^-^CD117^-^ mouse prostate non-stem cells [2].

Therefore, in addition to the breast and pancreatic tissues, evidence from prostate cancer also indicate that androgen deprivation or other inducers may lead to an EMT phenotype accompanying with the acquisition of stem cell properties. Taken together, modulation of EMT may attenuate the stemness of CSCs.

### Signaling pathways involved in epithelial-to-mesenchymal transition and cancer stem cells in castration-resistant prostate cancer

Although EMT and CSC play a pivotal role in the development of CRPC, what mechanisms might be responsible for EMT or CSC-conferred castration resistance are not well understood. One key mechanism is likely related to the AR and AR signaling, a classic pathway leading to CRPC [1]. Besides the AR axis, the activation has also been presumed to be stimulated by some other pathways, such as growth-factor receptor tyrosine kinase (RTK) activated pathways, Pten related pathway, signal transducers and activators of transcription 3 (STAT3) related pathway, Wnt, Notch and Hedgehog signaling pathways [37-41].

#### AR pathways

The precise role of AR axis in CRPC and prostate cancer metastasis has been well recognized in the last decades. In addition to the commonly known AR amplification and AR mutant, recent studies have found that androgens and the androgen receptor are functionally required in the process of EMT and the maintenance of prostate stem/progenitor cells. To some extent, androgens can induce EMT in PC-3 and LNCaP prostate cancer cells, with reduced epithelial marker expression and increased level of mesenchymal marker. Androgens alone or in combination with TGF-β enhance the capacity of prostate cancer cell migration and invasion, with a significant increase in Snail expression. Meanwhile, only low level of AR is required in androgen-induced EMT phenotype alteration [42]. Via a possible AR-Zeb1 bidirectional, negative feedback loop, Sun et al [2] have found that androgen deprivation could lead to EMT in both normal prostate and prostate cancer tissues. In addition, Slug, another EMT transcription factor, could cooperate with AR and promote the development of CRPC [11]. A recent study reveals that mucin 1 (MUC1) C-terminal subunit (MUC1-C) could form a complex with AR, which could not only occupy the PSA promoter but also associate with induction of the EMT modulated by miR135b mediated [43] or ZEB1 mediated mechanism [44]. Moreover, MUC1-C overexpression in androgen sensitive LNCaP cell also increases cell growth following androgen depletion and anti-androgen (such as bicalutamide) treatment, implicating its role in the occurrence of CRPC. Furthermore, the methylation in CpG islands of AR promoter is likely related to prostate stem/progenitor cells stemness and differentiation. As a result, prostatic epithelial cells, PCSCs and LNCaP progenitor/stem cells present low AR expression due to high DNMT1/3 level and MBD2-AR promoter binding. Moreover, treatment of prostate cancer cells with 5-AZA, a specific DNA methylation inhibitor, results in an inhibition of self-renewal/growth of prostate stem/progenitor cells *in vitro* and reduces prostate tumorigenicity *in vivo*[45].

#### Growth-factor receptor tyrosine kinase (RTK) activated pathways

Progression of prostate cancer to CRPC is also associated with enhanced expression of growth factors and receptors capable of activating the receptor tyrosine kinase (RTK) pathways. Utilizing the androgen refractory carcinoma of the prostate (ARCaP) cell model, Graham et al investigated the effect of IGF-I on ZEB1 expression. The ARCaPM (M = mesenchymal) cells show higher expression of ZEB1 than the ARCaPE cells (E = epithelial). IGF-1 treatment up-regulates the mRNA and protein levels of ZEB1 *in vitro* via activation of the MEK/ERK pathway. Furthermore, treatment of prostate cancer cells with ZEB1 siRNA results in a more epithelial morphology, with increased expression of E-cadherin and decreased N-cadherin, fibronectin expression, and suppresses prostate cancer cell migration and invasion. These results together suggest that IGF-1 is a key regulator of EMT in prostate cancer, which induces cell invasion, metastasis and CRPC [8]. Using an inducible FGFR1 (iFGFR1) prostate mouse model, Acevedo et al have found that iFGFR1 activation by chemical inducers of dimerization (CID) results in prostate adenocarcinoma that is closely associated with EMT, while CID withdrawal causes a full reversion of PIN. iFGFR1-induced prostate cancer presents higher nuclear EMT-associated Sox9 expression and liver and lymph node metastases [38]. As a part of general signaling activities for most growth factor receptors, activation of endogenous c-Ras might be an important mechanism for CRPC. For example, induced RasN17 expression (a dominant negative form of Ras) in C4-2 cell line increases the sensitivity to Casodex (an anti-androgen drug), inhibits cell proliferation *in vitro* and causes a dramatic regression of C4-2 xenografts after surgical androgen ablation *in vivo*[46].

#### Pten related pathways

Pten loss and the activation of Pten/PI3K/AKT have been well understood in prostate tumorgenesis and its progression to castration resistance, indicating that Pten related pathway may play an important role in CRPC. Higher level of p-MAPK is observed in malignant and CRPC prostate tissues than non-malignant specimens [37]. Using a series of prostate mouse models, Mulholland et al [37] have reported that in contrast to Pb-Cre+;PtenL/L and Pb-Cre+;K-rasL/W, prostate samples from Pb-Cre+;PtenL/L;K-rasL/W mutants present an EMT phenotype, with increased expression of mesenchymal molecules including Vimentin, Fibronectin, Snail, Twist and Zeb1. Thus, activation of RAS may contribute to the development of EMT in Pten-deletion prostate epithelial cells. As it is mentioned above, recent studies show that EMT is mechanistically associated with the acquisition of cancer stem cells. The Pten;K-ras prostates, which also present a remarkable expansion of LSC^hi^ subpopulation, have enhanced sphere-forming ability compared to Pten deletion prostates. Further isolation by FACS show that only Lin^-^EpCAM^low^CD24^low^ cells from C+;PtenL/L;K-rasL/W mutants gain mesenchymal properties accompanied by higher sphere-forming capacity. The EpCAM^low^/CD24^low^ subpopulation displays mesenchymal cells signatures, with enhanced level of AR and mesenchymal cell markers [37]. Therefore, activation of RAS/MAPK pathway may function as a second hit following changes of the well-known PTEN/PI3K/AKT pathway to androgen-insensitive prostate cancer and CRPC [37].

#### STAT3 related pathway

Comparing hormone-naive tissue samples with CRPC specimens, Rocchi et al [47] find that molecular chaperone Hsp27 mRNA and protein level are increased after ADT. Over-expression of Hsp27 in LNCaP cells displays highly resistance to anti-androgen reagents *in vitro* and *in vivo*. Compared with mock-transfected controls, tumor volume and serum PSA levels are dramatically increased after castration in LNCaP-Hsp27 tumors, suggesting that increased Hsp27 levels can promote the development of CRPC. Furthermore, treatment with Hsp27 antisense oligonucleotides (ASO) or HSP27 shRNA can lead to an inhibited proliferation in LNCaP cells, induce apoptosis via inhibition of STAT3 activity *in vitro* and reduce the capacity of tumorgenesis after castration *in vivo*. These results have implied that Hsp27 might be a regulator of STAT3-induced apoptosis in the condition of androgen ablation and be a promising therapeutic target in CRPC [47]. Another report about Hsp27 from their lab indicates that Hsp27 over-expression can regulate EMT in prostate cancer, accompanied by a mesenchymal cell morphology switch. While Hsp27 promotes EMT with enhanced cell migration and invasion ability, silencing Hsp27 could reverse these EMT phenotypes, with reduced STAT3 phosphorylation and its binding to the Twist promoter. These observations suggest that instead of inducing apoptosis, Hsp27 also functions as a key regulator for IL-6–induced EMT via STAT3/Twist signaling pathway [39]. A recent study from Collins and colleagues has explored the contribution of STAT3 signaling pathway to prostate stem/progenitor cells [48]. Treatment with either specific IL-6 antibody or a specific pSTAT3 inhibitor (LLL12), leads to a reduced colony-forming capacity of the stem-like cells from a high-grade clinical prostate cancer sample. Using a murine xenograft model derived from a castration-resistant patient with generally activated STAT3, they found that cells from the xenografts show dramatically decreased tumorigenicity when treated with LLL12. These results suggest that blocking STAT3 might be a novel strategy in the future to suppress tumor initiation capability of human prostate cancer [48].

#### Wnt pathways

In addition to the pathways mentioned above, alterations in the Wnt, Notch and Hedgehog pathways have also been reported to contribute to formation of CRPC. Based on β-catenin immunocytochemical analysis, Wan et al [49] have revealed the functional role of Wnt/β-catenin in CRPC. In their study, high levels of β-catenin is observed in human prostate cancer tissues, which is inversely linked with AR expression, raising the possibility that low or no AR expression activates Wnt/β-catenin signaling. This evidence may explain the phenomenon that only low level of AR lead to androgen-induced EMT phenotype [42]. A recent study reports that Sox2 promotes EMT via activation of WNT/β-catenin, with improved migration and metastasis ability *in vitro* and *in vivo*. Over-expression of Sox2 in human breast cell line-MDA231 and human prostate cancer cell line DU145 causes enhanced migration capacity and decreased levels of E-cadherin, DKK3 and increased a-SMA, DVL1 and DVL3. Further experiments indicate that Sox2 promotes EMT via binding to the promoter of β-catenin [50]. Additional studies find that the promoter of WIF1 (a WNT inhibitor) is hypermethylated, resulting in its down-regulation in most prostate cancer cell lines [51]. Consistent with the WIF1 over-expression experiments, restoration of WIF1 by treating the cells with 5-Aza induces MET, a reverse process of EMT, with upregulation of epithelial markers (E-cadherin, CK8/18), down-regulation of mesenchymal markers (N-cadherin, Fibronectin, Vimentin, Slug and Twist) and decreased activity of MMP-2/9 *in vitro*. Meanwhile, over-expression of WIF1 dramatically reduces tumor growth in a xenograft mouse model, accompanied by an increased E-cadherin and CK18 expression and a decreased vimentin level in tumor tissues [51]. Moreover, WNT inhibitors reduce the sphere-forming and self-renewal ability of prostate cancer cells. The opposite results are observed when the cells are treated with WNT3a, with an increased expression of β-catenin, CK18, CD133 and CD44 [51]. Therefore, WNT signaling affects the functions of CSCs [40].

#### Notch and hedgehog pathways

As an important regulator of cell fate determination, Notch signaling is reported to play crucial roles not only in prostate development but also in the progression of prostate cancer. Recent studies have suggested that Notch pathway elements are positively participated in both normal and malignant prostate stem/progenitor cells [52]. Similar to this observation, inactivation of Notch1 dramatically suppresses the clonogenic and prostasphere forming ability, implying that Notch1 is at least partially responsible for maintaining the prostate stem/progenitor cells. Expression of Notch1 is reported to be mediated by miR-200b and miR-200c, which links CSCs signatures with EMT phenotypes. However, further studies are required to unveil whether other Notch pathway elements regulate cancer stem/initiating cell within the prostate [36]. It has been well established that the Hedgehog (Hh) signaling pathway plays central roles in developmental patterning and regeneration of prostate epithelium. Treatment with cyclopamine, a specific inhibitor of the Hh pathway, suppresses tumor growth at 10 mg/kg and actual regression at 50 mg/kg in established PC3 and 22RV1 xenograft tumors *in vivo*[41]. Moreover, cyclopamine suppresses transcription of the gene encoding nestin and the Polycomb group protein Bmi-1, two stem cell markers, indicating the role of Hh pathway activity in prostate progenitor cells. Using the castration-regeneration cycle model, Hh signaling pathway blockade by cyclopamine leads to loss of regenerative capacity in rodent ventral prostates. Examination of levels of Hh pathway targets PTCH and GLI in metastatic and benign prostate tissues shows that Hh pathway activity is strongly correlated with prostate cancer metastasis, which is attributed to the involvement of EMT [41].

Taken together, these results suggest that all the pathways mentioned above can either function alone or in combination with each other during prostate cancer progression. The advanced CRPC is linked with EMT and/or CSCs, revealing a possible mechanism in the transition of prostate cancer to an androgen-independent state. Drugs designed to target these pathways may provide a promising direction for the future treatment of CRPC.

### Regulation of epithelial-to-mesenchymal transition activity, stemness of cancer stem cells and specific signaling pathways in castration-resistant prostate cancer

After recognizing the importance of EMT, CSCs and related signaling pathways during the development of CRPC, what we should do is to find a way to control these elements by either reversing or inhibiting the activation of these components so to prevent or alleviate CRPC. Studies thus so far suggest that inhibition of any one of these elements or multiple elements together are helpful in alleviation of CRPC.

A promising example is the study on N-cadherin and N-cadherin antibodies. As described above, Reiter and colleagues have reported that N-cadherin could cause metastasis and castration resistance of prostate cancer, but antibodies blocking N-cadherin not only delay the progression of prostate cancer to castration resistance but also inhibit invasion, metastasis and castration-resistant tumor growth *in vitro* and *in vivo*[12]. These data warrants that N-cadherin antibodies be validated in preclinical and clinical testing to determine their general toxicity. Therefore, antibodies directly against cell surface markers of EMT and/or CSCs might be a reasonable way to control EMT and CRPC. Another way to control EMT would be to use its related proteins and pathways. OGX-427, which currently in phase II trials by OncoGenex Pharmaceuticals, is another great example. Heat shock protein 27 (Hsp27) induces IL-6 dependent and independent EMT in prostate cancer by promoting phosphorylation and nuclear translocation of STAT3, making STAT3 to bind to the Twist promoter, and activating Twist function. Shut down of Hsp27 reverses EMT and decreases migration, invasion, and matrix metalloproteinase activity of prostate cancer cells. As an anti-sense inhibitor, OGX-427 suppresses Hsp27 and reduces circulating tumor cells and tumor metastasis [39]. Another group also reports that regulators of EMT have a good effect on several types of cancers (ovarian, nasopharyngeal and esophageal carcinomas), especially in prostate cancer [53]. Chu and colleagues have applied two water-soluble contents of garlic, S-allylcysteine (SAC) and S-allylmercaptocysteine (SAMC), to suppress proliferation and invasion of androgen-independent prostate cancer. The inhibitory effect appears to be due to the reverse of EMT: mesenchymal to epithelial transition (MET). During the reverse transition, E-cadherin is restored and activated whereas Snail expression is decreased in prostate cancer cells [53]. One more evidences of targeting EMT is the study of NPI-0052 by Baritaki and colleagues [54]. They show that the proteasome inhibitor NPI-0052 reverses castration resistance to androgen sensitive via inhibiting EMT in human prostate cancer cell lines. NPI-0052 regulates NF-κB-Snail-RKIP pathway by suppressing NF-κB inhibition, down-regulating the EMT biomarker Snail, and up-regulating Raf-1 kinase inhibitory protein (RKIP). Snail is a crucial target for reversal of resistance. Further experiments indicate that administration of NPI-0052 leads to inhibit anti-apoptotic gene and metastasis [15,54].

On the other hand, molecules targeting cancer stem cells are investigated. Liu and colleagues have demonstrated miR-34a as a potential therapeutic target against prostate cancer stem cells [55]. CD44^+^ prostate cancer cells are purified from xenografts in mice and primary tumors in humans as prostate CSCs. They display enhanced tumor proliferating and metastatic capacities in CD44^+^ population. MiR-34a is down-regulated in the CD44^+^ cells. Up-regulation of miR-34a leads to an inhibition of sphere formation and tumor progression in prostate cancer cells and in CD44^+^ cell. The study of miR-34a indicates that a critical negative regulator of CSCs could be an attractive therapeutic option in prostate cancer with a cancer origin of stem cells. Similarly, another negative regulator of prostate cancer stem cells is identified by Kroon and colleagues [48]. They demonstrate that pSTAT3 is expressed specially in prostate stem-like and progenitor cells with co-expression of IL-6 receptor gp80. If LLL12, a novel pSTAT3 inhibitor, is applied to prevent STAT3 from phosphorylation, the stemness of prostate cancer cells is inihibited, resulting in significant reduction of cancer cell proliferation in primary cultures from patients with high Gleason grades. Strikingly, blocking pSTAT3 by LLL12 also abolishes the outgrowth of castrated tumors from patients in a mouse xenograft model. In addition, targeting the STAT3 pathway in prostate CSCs might be a promising therapeutic way by another group. Hellsten and colleagues [56] find that ALDH + prostate cancer cells are cancer stem cell-like cells as they display the properties of CSCs such as self-renew, clonogenicity and tumorigenicity as well as elevated expression of CD44 and integrin α2β1, two CSCs markers, and pSTAT3. Besides galiellalactone, a direct inhibitor of STAT3 pathway, in the culture medium, suppresses proliferation of ALDH + cells. These findings emphasize that targeting CSCs in prostate cancer is a considerable therapeutic approach.

In addition to directly target EMT and CSC, potential therapies targeting signaling pathways related to EMT and CSC are also investigated. Mulholland and colleagues apply mTOR inhibitor rapamycin and MEK inhibitor PD325901 to target the PI3K/AKT and RAS/MAPK signaling in an *in vivo* prostate cancer model using a bioluminescence image monitoring method. The combined treatment decreases EMT phenotypes, cell proliferation, and metastasis of the C+;PtenL/L;K-rasL/W; Rosa26-luc sphere cells to the lung or the metastasis produced by stem/progenitor cells purified from C+;PtenL/L;K-rasL/W transgenic mice [37]. It is widely believed that castration enriches stem/progenitor cells in the prostate. Using the castration and androgen replacement prostate regeneration paradigm, Hh pathway blockade by cyclopamine or Hh-neutralizing 5E1 antibody dramatically attenuates prostate regeneration [41], implying that these Hh pathway inhibitors impede the function of prostate stem cells.

## Conclusions

In summary, castration resistance is a major clinic problem. In addition to the classic AR signaling, recent studies have present a large body of evidence that EMT [57-75], CSCs [76-86] and specific signaling [87-104] play important roles during the development of CRPC. Initial experiments also suggest that there is a link between EMT and CSCs. EMT appears to be a 2-way dynamic process. It is proposed that while EMT may enhance the stemness of CSCS and enhance the CRPC, reversing the EMT or MET may attenuate the stemness of CSCs and alleviate CRPC. Further studies will reveal more molecular mechanisms involved in CRPC. It is also possible that these mechanisms can function either alone or in combination with each other.

Nevertheless, focusing on the axis of EMT, CSCs and specific signaling pathways is a novel, breakthrough thinking in the war against CRPC. Among them, development of antibodies against the surface biomarkers including those EMT markers such as N-cadherin, CSC markers such as CD133, and specific signaling molecule such as HSP27 is the easier approach than targeting the transcription factors or cytoplasmic molecules and might hold promise for a novel therapeutic approach for the treatment of prostate cancer. Clinical trials of molecules in these categories to rule out toxicity and to demonstrate efficacy are required to achieve this goal.

## Abbreviations

AR: Androgen receptor; EMT: Epithelial-to-mesenchymal transition; CSCs: Cancer stem cells; CRPC: Castration-resistant prostate cancer; ADT: Androgen deprivation therapy; MET: Mesenchymal-epithelial transition; PIN: Prostatic intraepithelial neoplasia; PSA: Prostate specific antigen.

## Competing interests

The authors declare that they have no competing interests.

## Authors’ contributions

PL wrote the manuscript and prepared the Figure and Table, RY assisted in the preparation and WQG edited and modified the manuscript. All authors read and approved the final manuscript.

## Authors’ information

To become an Oncologist, Dr. Ping Li has studied as a doctor at Shanghai Jiao Tong University School of Medicine. Dr. Ru Yang is an Assistant Research Scientist in the Department of Stem Cell Research Center, Renji Hospital, Shanghai Jiao Tong University School of Medicine. Dr. Wei-Qiang Gao serves as a professor and Director of State Key Laboratory of Oncogenes and Related Genes and a professor and Director of Stem Cell Research Center, Renji Hospital, Shanghai Jiao Tong University School of Medicine, Dr. Gao also serves as Chair Professor and Associate Dean of Med-X Research Institute Shanghai Jiao Tong University. Dr. Gao has a long-standing interest in the field of cancer research and developmental neurobiology and published papers as the corresponding or first author in journals including Nature, Cell, Science, PNAS, Cancer Research, Neuron, Nature Neuroscience, Development, etc.
